# Perspectives of health service providers in delivering best-practice care for Aboriginal mothers and their babies during the postnatal period

**DOI:** 10.1186/s12884-022-05136-6

**Published:** 2023-01-05

**Authors:** Jocelyn Jones, Angela Durey, Natalie Strobel, Kimberley McAuley, Karen Edmond, Juli Coffin, Daniel McAullay

**Affiliations:** 1 National Drug Research Institute, Curtin University, WA Perth, Australia; 2 School of Population and Global Health, The University of Western Australia, WA Perth, Australia; 3 Kurongkurl Katitjin, Edith Cowan University, WA Perth, Australia; 4 King’s College London, London, UK; 5 Telethon Kids Institute, WA Perth, Australia

**Keywords:** Aboriginal, Mothers, Postnatal care, Best practice

## Abstract

**Background:**

Evidence suggests that Aboriginal babies in Western Australia are not receiving adequate primary health care in their first 3 months of life, leading to questions about enablers and constraints to delivering such care. This paper presents findings from a qualitative research project investigating health providers’ perceptions and experiences of best and current practice in discharge planning, postnatal care and health education for Aboriginal mothers and their newborn babies.

**Methods:**

Constructivist grounded theory guided this research involving 58 semi-structured interviews conducted with health providers who deliver care to Aboriginal mothers and infants. Participants were recruited from hospital-based and primary health sites in metropolitan Perth, and regional and remote locations in Western Australia.

**Results:**

Structural factors enabling best practice in discharge planning, postnatal care, and health education for mothers included health providers following best practice guidelines and adequate staffing levels. Organisational enablers included continuity of care throughout pregnancy, birth and postnatally. In particular, good communication between services around discharge planning, birth notifications, and training in culturally respectful care. Structural and organisational constraints to delivering best practice and compromising continuity of care were identified as beyond individual control. These included poor communication between different health and social services, insufficient hospital staffing levels leading to early discharge, inadequate cultural training, delayed receipt of birth notifications and discharge summaries received by Aboriginal primary health services.

**Conclusion:**

Findings highlight the importance of examining current policies and practices to promote best practice in postnatal care to improve health outcomes for mothers and their Aboriginal babies.

## Background

Despite Australia’s wealth and investment in health, being unable to deliver consistent and effective health services to many vulnerable Aboriginal and Torres Strait Islander children is a significant concern [1]. Given the success of many Aboriginal led health programs [2], the 2021 Closing the Gap campaign (CTG) report into disparities in health outcomes between Aboriginal and non-Aboriginal Australians [3] reiterates the need for governments to share decision making with Aboriginal and Torres Strait Islander (hereafter Aboriginal) leaders and communities. Included in these recommendations is working together in early childhood care and development to ensure health and wellbeing outcomes improve. Recent Prime Ministerial reports on CTG specify that improvements in Aboriginal children’s health outcomes remain inconsistent [4, 5]. The 2020 report indicates that, while the health of Aboriginal mothers and children has improved over the last decade, the reduction in child mortality (7%) was not strong enough to reach the target of halving the gap [6]. It is important to recognise the ongoing legacy of colonisation that continues to manifest in the health and wellbeing of First Nation peoples. Australia’s history colonisation by the British in 1788 saw Aboriginal people (hereafter Aboriginal) dispossessed of their culture with their lands taken by white settlers. Apartheid-like policies of racial segregation and assimilation led to the forced removal of Aboriginal children from their families, where these children became known as the Stolen Generations. Such policies and practices have led to ongoing intergenerational trauma and grief for Aboriginal people reflected in, and compounded by, the current high rates of contemporary removal of Aboriginal children [7, 8]. Aboriginal children continue to be at significantly greater risk of involvement with government child protection services with child removal an ever-present threat [8, 9]. This history has led to Aboriginal mothers, not only being mistrustful of and hesitant to access mainstream services, but also fearful of attracting the interest of authorities which could lead to losing their children [10, 11].

It is well documented that personal and institutionalised racism is a ‘key social determinant of health for Aboriginal and Torres Strait Islander people’ and a ‘key driver of ill-health’ in the National Aboriginal and Torres Strait Islander Health Plan (NATSIHP) 2013–2023 [12]. In 2014, the Royal Australian College of General Practitioners released a position statement on racism in the healthcare system, recognising that “a spectrum of racist attitudes and behaviours are present” including verbal or behavioural abuse, unconscious bias, and institutional racism exist in society and in the Australian healthcare system. Racism in healthcare results in ‘compromised quality of medical care’, including type of treatment offered and lower uptake of essential care [13]. Addressing these issues systemically, organisationally and inter-personally needs to be a priority if health outcomes are to improve. Australian Aboriginal children still carry an unacceptably high burden of ill health [14, 15]. At best, programs for Aboriginal mothers show some improvements in antenatal care and pregnancy smoking rates [16]. Tertiary and primary care health services play an essential role in ensuring that quality care is provided to Aboriginal mothers during the antenatal, birthing and postnatal period to mitigate risk factors. However, the Aboriginal community is concerned that over 50% of Aboriginal babies in Western Australia are not receiving the primary care they need in the first 3 months of life, including immunisation, despite being at their most vulnerable and having high rates of hospitalisation [4, 14, 15, 17–19]. Hospital admissions are two-fold higher in Aboriginal compared to non-Aboriginal babies over this period, and emergency department presentations are even higher [20–22]. Aboriginal child deaths occurred mainly in the first year of life, most during the perinatal period [14, 15]. This is cause for significant concern. Ongoing and coordinated care is essential from the outset of an infant’s life and offers a safeguard that extends well beyond infancy and into their early childhood years.

Access to quality medical care, public health initiatives and safe living conditions are known protective factors that increase the chances of having a healthy baby. However, less is known about factors specific to postnatal care [16]. A key contributor to positive perinatal outcomes for Aboriginal mothers is culturally secure care [23]. Culturally secure care is demonstrated when a health practitioner builds their own understanding and awareness of Aboriginal culture and applies it to their practice, so Aboriginal people feel respected and safe to access health care. This is further strengthened when the policies and procedures of an organisation or service also reflect these principles [23, 24].

Primary health services (defined here as community-based health services, including maternal and child health care) can significantly impact the health of Aboriginal babies over the period of early infancy [17, 18]. Evidence demonstrates the efficacy of primary care services providing home visiting and prevention strategies for mothers and babies to improve child health outcomes [15–18]. Maternal and child health nurses are the conventional service providers in this area, yet Aboriginal families mainly attend other services such as Aboriginal Community Controlled Organisations (ACCHO^1^), Primary Health Networks and general practice surgeries, which are not specifically trained in maternal and child health [1]. These services do not routinely receive state provided birth notifications and hospital discharge summaries due to concerns about confidentiality and consent [1]. This raises questions about the continuity of care from hospital to home, where discharge information may not reach the relevant primary health providers resulting in a lack of coordination in the ongoing care of mothers and their babies [1]. This situation is compounded when families move between houses, regions, districts and jurisdictions, making it difficult to link with primary care providers [15, 25, 26].

The research question guiding this paper is ‘What enables or constrains best practice in delivering postnatal care to mothers and their Aboriginal babies in the first three months of life?’ The paper presents findings from the qualitative aspect of a population-based randomised control trial. The trial is a behaviour change intervention to test the effectiveness of a new model of early infant primary care coordination to improve health and access to primary care for Aboriginal infants aged under 3 months old in Western Australia [1]. The qualitative project investigated primary health care providers’ perceptions and experiences of best and current practice in discharge planning, postnatal care and health education for mothers and their Aboriginal newborns. This paper provides evidence of what enabled or constrained best practice in these areas and offers participants’ suggestions of how to improve care, noting similarities and differences within and between sites.

## Method

Semi-structured interviews were conducted with health providers in hospital-based sites and non-government and government primary health care services in Perth, regional and remote locations in Western Australia. A social constructivist approach was chosen as it seeks to explain reality as constructed by individuals within a particular context. Participants’ voices and interpretations in their interactions with the researcher are integral to examining the phenomenon of interest [27]. Constructivist Grounded Theory (CGT) seeks to generate new theory based on data gathered through interviews and focus groups. CGT is adaptable and can connect ‘…cultural gaps and chasms between the cultures of methodological origination and application’ [28]. The inductive approach guides this research, given the need to better understand health providers’ perspectives and experiences of the enablers and constraints to delivering best practice in discharge planning, postnatal care and health education for mothers and their Aboriginal babies in Western Australia. This method enabled the researchers to interact with, interrogate, and reconstruct the data by going beyond the surface of participants’ responses to seek the deeper meanings, including understanding factors contributing to gaps in infant care coordination [29, 30].

### Study setting

Data collection for this study was undertaken in from 2016 to 2019. The research was conducted at five hospital sites (two metropolitan, one regional, two remote) and five government and non-government primary health care sites (three metropolitan, two remote) (Table 1).

**Table 1:** Research sites

Site	Code
Metro- hospital	Metro1H
Metro- PHC	Metro1C
Metro- PHC	Metro2C
Metro- hospital	Metro3H
Metro - PHC	Metro3C
Regional- hospital	Reg4H
Remote - hospital	Rem5H
Remote- PHC	Rem5C
Remote- hospital	Rem6H
Remote- PHC	Rem6C

Interviews were conducted by three senior Aboriginal researchers in a private, quiet space or room located in the hospital or primary health care site.

### Recruitment of participants

Directors and managerial staff from the intervention hospitals and primary care services were approached individually with information about the study to gain their support and secure participation. Primary health care staff working in maternal and child health within these services were purposively recruited to participate in an interview. Staff included midwives, nurses, administration staff, Aboriginal Health Workers (AHW) and doctors.

All participants involved in the interviews were initially given an information sheet outlining the study and conditions of participation, including an opportunity to ask any questions before providing written consent. They were also advised that they could decline to answer a particular question and could withdraw from the interview at any time without needing to give a specific reason.

### Data collection

Data were collected through semi-structured, face-to-face interviews or telephone interviews when face to face interviews were not possible. All interviews were audio-recorded and transcribed verbatim, and transcripts were de-identified before being imported into NVivo (http://www.qsrinternational.com) to help organise and manage the data.

### Data analysis

Guided by constructivist grounded theory [29, 30] an iterative approach to analysis was taken. An Aboriginal epidemiologist with expertise in qualitative data collection and analysis and an experienced qualitative researcher open coded the interview transcripts. This initial step involved the development of codes which researchers used to identify core categories when analysing and interpreting the data [31, 32]. This process was further refined through axial coding, where categories identified through the codes were interrogated to note any links or relationships between them [32]. Memos were also recorded based on the researchers’ reflections on the data during the process of analysis and interpretation [29, 30]. Theories are attained by researchers constantly analysing and comparing data and interrogating and comparing interpretations of the data that they have translated into codes and categories [29, 30, 32]. Rigour was further established through discussion and review of findings and interpretations with co-researchers. Categories were identified and agreed upon through discussions relating to the project’s aims, specifically noting similarities and differences in participants’ responses.

### Ethics

This study was approved by the Western Australia Aboriginal Health and Ethics Committee (WAAHEC) and the Department of Health Western Australia ethics committees.

## Results

Fifty-eight interviews were conducted with midwives, AHWs, child health nurses, a General Practitioner and an Aboriginal Grandmother Liaison Officer. To facilitate comparison between sites, Table 2 identifies the location from which the quotes were taken.

**Table 2:** Participant IDs (de-identified)

Site	Code	Participants	IDs
Metro – hospital	Metro1H	7	Metro1H:1–7
Metro – community	Metro1C	2	Metro 1C:1–2
Metro – community (non-government)	Metro2C	4	Metro 2C:1–4
Metro – hospital	Metro3H	8	Metro 3H:1–8
Metro – community	Metro3C	6	Metro 3C:1–6
Regional – hospital	Reg4H	10	Reg 4H:1–10
Remote – hospital	Rem5H	9	Rem 5H:1–9
Remote – community (non-government)	Rem5C	3	Rem 5C:1–3
Remote – hospital	Rem6H	8	Rem 6H:1–8
Remote – community (non-government)	Rem6C	1	Rem 6C:1
**Total**		**58**	

Participants’ perspectives and experiences of enablers to best practice in discharge planning, postnatal care and health education for mothers and their newborn babies across sites are summarised initially, followed by perceived constraints in current practice to delivering best practice in these areas. Findings are organised into structural and organisational categories. Structural factors include broader political, socioeconomic and environmental conditions and institutions that either facilitate or constrain best practice [33]. Organisational factors consist of elements in a workplace that affect the people who work with policies and procedures [12]. Under each category, key themes are identified. Finally, participants’ ideas about addressing these constraints are described under the subheading ‘Suggestions to facilitate best practice.’

### Enablers to best practice in discharge planning, postnatal care and health education

#### Structural factors

National and State Best Practice Guidelines supported hospital staff in discharge planning to ensure continuity of care from early pregnancy through antenatal to postnatal care. According to participants in the hospital sites, discharge planning involved checking best practice guidelines to ensure the planning and health education initiated during early pregnancy continued throughout the antenatal and postnatal periods. Best practice was enabled through adequate staffing levels to ensure continuity of care. Ideally, the same midwives were present throughout pregnancy, birth and the postnatal period to build relationships, trust and confidence with the mother.

### Organisational factors

#### Continuity of care

While there was overlap between health education and postnatal care, all participants highlighted various elements of best practice. These included completion of satisfactory observations on mother and baby and ensuring the mother felt empowered and capable of looking after her baby on discharge.

“Okay, so what should happen is that the mum will go home feeling confident with her breastfeeding, her baby care and being able to recognise any signs of infection in herself and with her baby. Also have a good understanding of safe sleeping for babies, safe transport … seeing what her social supports are and making sure that she’s got a good network. Also, refer to child health nurses … or another agency in the community” (Metro 3H:3).

Participants from primary health services commented that communication and collaboration between the hospital and their service facilitated a smooth transition from hospital to community-based care. This included the timely distribution of birth notifications and discharge summaries to facilitate ongoing best practice in postnatal care.

Several participants across sites spoke of woman-centred holistic care, where mothers were asked what they needed to be confident looking after their baby. This care also included support for breastfeeding or bottle feeding and informing mothers about risk factors such as Sudden Infant Death Syndrome (SIDS) and the need for safe sleeping. Adequate time for hospital staff to deliver best-practice was important. In one smaller metropolitan hospital with less pressure on early discharge and making beds available for new admissions, participants were often able to give more time and attention to new mothers who were able to stay in the hospital longer.

Given the importance of continuity of care, staff checked appropriate documentation was completed prior to discharge and gave the mother the ‘purple book’ as a printed resource to support her postnatal care once she went home. It was provided by the Health Department of WA usually at the maternity hospital and recorded the child’s health, growth and development appointments with health professionals from birth to school entry.

### Effective communication

A key element of best practice was staff communicating with the mother clearly and respectfully in ways that built the mothers’ confidence and trust. This included checking she understood instructions on caring for herself and her baby before going home and that family support was available on discharge. The ‘purple book’ also included reminders about follow-up appointments, immunisations and key contact details of services in case of need.

“Well, just making sure that the women feel that when they leave here, they’re very capable of looking after their baby in a safe, appropriate manner. That they’re bonding, that they feel good about feeding and bathing and general baby care, and that they know where to go if they find they don’t feel good. Where they can seek help” (Reg 4H:9).

### Constraints to best practice in discharge planning, postnatal care and health education

While participants aspired to deliver best practice in discharge planning, postnatal care, and health education to mothers and babies in their care, many felt frustrated by structural and organisational constraints beyond their control that inhibited delivery.

### Structural barriers

Structural barriers to delivering best practice included inadequate staffing levels, early discharge, no access to birth notifications or discharge summaries, and limited collaboration between health and social sectors in postnatal care.

### Early discharge

While many mothers wanted to go home as soon as possible after giving birth, some participants in metropolitan and remote sites felt early discharge wasn’t always ideal given the limited time for postnatal health and education, particularly when the ward was busy. This could lead to the mother being inadequately prepared to confidently look after her baby.

“I think it’s wrong that we are pushing these mothers’ home with their babies …when they know nothing about having a newborn baby” (Metro 1H:2).

Participants from regional and metropolitan sites expressed frustration at hospital management’s focus on early discharge and perceived lack of understanding of midwifery concepts around best practice.

“I don’t think postnatal care is done very well at all anymore unfortunately. I mean that - and it’s probably a bit of a consequence of hospitals being under so much pressure to get mums and babies out early” (Metro 1C:2).

This was compounded by inadequate staffing levels available to deliver high-quality postnatal care.

### Accessing birth notifications and discharge summaries

Postnatal care was also compromised when birth notifications and discharge summaries were not sent to primary health care services involved in the mother’s care. In Western Australia, the Department of Health clinical perinatal database ‘Stork’ has all public hospitals based in Western Australia inputting birth data within 24 hours of birth. These notifications are provided to government health services within 48 hours of birth. However, they aren’t available to non-government primary health care services. Several participants from non-government primary health services were frustrated at being excluded from being able to access these data and appropriate discharge summaries:

“We don’t get any notification and then the only way that we see that information is when we go out to see the families and, majority of the times, the printout is in the baby’s purple book, and so then we take that and then we will contact the government child health nurses and say, ‘we’re linked in with this family’” (Metro 3C:5).

“We’ve had mums that have had babies and we’ve had no idea, and we only find out because we run into them and then we’re like, ‘oh, you’ve had another one’, and then you kind of miss those first few checks” (Metro 2C:1).

One midwife at a metropolitan non-government primary health service had no access to STORK data and commented the service only received “about 40-45% of discharge summaries” (Metro 2C:3). The majority went instead to the GP or maternal child health team. While smaller hospitals in the metropolitan area seemed to liaise better with non-government primary health services (Metro 2C:3), discharge information about what had happened to mothers from other hospitals was difficult (Metro 2C:2).

Not having access to the data in remote areas was a significant issue for non-government primary health services as it compromised ongoing postnatal care:

“There is an issue with not having access to Stork because I’m not a WACHS (Western Australian Country Health Service [government]) employee, I’m not permitted access to Stork so therefore I don’t get the discharge summaries and that’s a big obstacle … The doctors here get sent Stork and discharge summaries. I don’t know it’s particularly reliable quite honestly. Because the pharmacist has access to those summaries, she forwards them onto me, so it’s a very messy system. I need access to Stork, that’s a real problem. (Rem 5C:1).

Participants commented that excluding their services from access to the Stork system could negatively impact post-discharge care for a mother and baby, particularly if they return to a remote area.

“There can be a delay from their part in sending the GP discharge summary. Whereas the lady might have arrived back in the [remote area] and we don’t have any information on her. … It’s very difficult to get that sent directly to us, the discharge summary for mum and babes for our high-risk clients” (Rem 6C:1).

### ‘Siloed’ approach to postnatal care

Participants in many sites discussed the lack of collaboration between sectors. Services act in ‘silos’ with little overlap or capacity for midwives to continue relationships with the mothers beyond the immediate postnatal period once the child health nurse took over. All participants were committed to supporting mothers as best they could to build their confidence and capacity to care for their babies. However, working with other sectors such as the Department of Child Protection (DCP), now Department of Communities, was often challenging. Several participants noted that some mothers lacked confidence in caring for their Aboriginal babies, which may be underpinned by fear.

“Am I good enough, am I a good enough mum, are you going to tell DCP on me? I’ve seen a lot of girls here absolutely nervous and needing to hear that they’re okay. Sometimes you get that little quiet voice who is very brave that actually says do you think I’m good enough as a mum? I say good enough for what? Good enough that DCP won’t come. That’s very heartbreaking” (Metro 2C:3).

One participant from a metro non-government primary health service noted that 70% of her caseload were connected to DCP, and yet:

“I think there is no mum that I’ve ever seen that does not want what’s best for their child, and I think what you see is that they actually don’t know what to do” (Metro 1C:1).

Participants noticed that mothers often felt vulnerable and scared, sometimes with good reason.

“The girl might be feeding her baby, come in the room. I’ve been in there; I’ve been in them situations. She’s said, ‘what are [youse[sic]] here for?’ They just came straight to her and took the baby off the tit. She started crying and I started crying. Police were in the next room. That’s heartbreaking. This has happened more than once. They just take the kids” (Metro 2C:1).

Participants from two metropolitan primary health services acknowledged the social challenges some mothers faced post-discharge, including overcrowded housing, alcohol and other drugs, and domestic violence. From a health and wellbeing perspective, lacking the basic needs of a stable home, money, and food security often meant the safety of mothers was compromised. This is heightened by the fear that DCP would take their babies away.

“That’s another thing that I have a little bit of a thing with DCP. If the last three kids have been taken away, then the new baby will also be taken away … Okay, give the mum a chance now to do the right thing and care for her baby. Be a mum and look after your baby” (Metro 2C:1).

Post-discharge is an essential period to provide adequate support for a mother. Therefore, clear communication and collaboration between services concerning prevention are paramount.

“I think that’s where I see that gap in Child Protection, that there’s no steps in place to actively help them keep their children; it’s more let’s step in when it’s at crisis point and just remove them, because that’s the only thing we can do. Whereas what I would like to see change … are we not at a point now where it’s becoming a crisis when our children are being removed, as if it’s, like, normal” (Metro 1C:1).

### Organisational barriers

Organisational barriers centred around themes related to a) lack of time and work pressures to deliver high-quality health education and postnatal care, often resulting from inadequate staffing levels in busy maternity wards; b) staff training in culturally responsive care and c) poor communication and collaboration between hospitals and non-government primary health services.

### Staffing levels

While midwives were expected to complete discharge planning documentation before mother and baby were discharged, participants from every site noted considerable barriers to achieving this on time. These included early discharge, heavy workloads, an abundance of paperwork and being short-staffed.

“It seems like we’re almost trying to push people out the door and there’s not always the opportunity to – take one simple thing like breastfeeding problems, quite often we’re getting rid of ladies out of here before their milk’s even come in, because it’s happened so quick. So how are we going to identify breastfeeding problems in that population if we’re getting rid of them before their milk’s even come in” (Rem 5H:6).

While health education also suffered from lack of time, several participants commented on the significant amount of information mothers received.

“But the thing is that a lot of it is overwhelming and there’s a lot of information, too quickly, too much” (Metro 3C:3).

In one metropolitan site where most mothers of Aboriginal babies discharge 4 to 6 h after giving birth (Metro 1H:7), an Aboriginal Grandmother Liaison Officer was on staff to visit mothers on a needs basis to support them. Some regional mothers were discharged up to 3 days after giving birth and followed up with home visits by the midwife (Reg 4H:5). In one remote site, agency nurses were often employed for a limited time if the ward was short-staffed. Orientation to the ward when they arrived included limited cultural training. Some completed the online cultural training course, while others attended locally organised cultural training sessions. Although it is a requirement of staff employed by the Western Australian government to have cultural training this is not the case for agency staff with some not having completed any cultural training.

### Health education

There was a significant focus on the health benefits of breastfeeding provided to mothers. However, some participants noted that many mothers changed to bottle-feeding soon after discharge. Various reasons were given, including grandmothers and aunties being able to help the mother with feeding. During education sessions with mothers breastfeeding was encouraged. However, midwives also provided advice to mothers about bottle-feeding. One participant felt that, in relation to breastfeeding, it was important to focus on woman-centred care and listen to the mother:

“…some of the midwives, their feelings overshadow what the mums want” (Reg 4H:1).

Safe sleeping was another significant area of health education, including discussions around co-sleeping to reduce the risk of SIDS.

While mothers were informed about the importance of follow up appointments, there were logistical constraints. Mothers often had limited access to transport following discharge or to access follow up appointments. Despite one site offering transport options for mothers, others didn’t, and this was identified as a barrier to follow up care and ran the risk of some mothers falling through the gaps.

“… access to our service, helping our clients to access. I think that’s one of the biggest things. It’s not that they don’t necessarily want to come in or anything, but I think access is difficult” (Metro 2C:4).

### Cultural training

An Aboriginal health practitioner in one remote site helped staff learn about Aboriginal culture. More generally, cultural training involved completing the mandatory online e-learning course. However, not all participants had completed it from a remote hospital, and others in a metropolitan hospital questioned its value, preferring in-person rather than online presentations. Others working in a remote site found the information was general rather than specific to local Aboriginal cultural groups, which would have been more relevant and culturally appropriate.

“A lot of people don’t understand the whole cultural side of it, and I think it would be good to have a, you know, set aside team involved in Aboriginal women’s care and their children’s care because that’s what they need, they need a group of people that they know well, they feel comfortable with, that see them and I just think that’s how we’re going to get better outcomes” (Metro 3H:6).

### Poor communication and collaboration between the hospital and Aboriginal primary health services

Several participants were frustrated and concerned at the lack of communication and collaboration between hospitals and primary health services. Primary health services often did not communicate with each other either.

### Recommendations to facilitate best practice

#### Structural

Participants’ suggestions focused on postnatal care delivery from both hospital and primary health care settings. They included the need for adequate resourcing and time for staff on maternity wards to deliver best practice in discharge planning, health education and postnatal care. They also included better coordination and prompt dissemination of birth notifications and discharge summaries to primary health services to ensure continuity of postnatal care.

“Yep, it would be being on the Stork system, being acknowledged by different government organisations, hospitals in particular, GP obstetricians, DCP – just working better and being acknowledged more” (Metro 3H:5).

All participants noted that better communication and closer contact with the tertiary maternity hospitals would facilitate best practice. One community-based participant intimated a lack of awareness in hospital staff of non-government primary health services.

“They need to know that we’re here, and I don’t think they do. Some might and some don’t. We say, I work at […]. Who are they? Where are they? What do they do? They wouldn’t have a clue. There’re a few things there that we need to follow up” (Metro 2C:1).

Strengthening relations and collaboration between hospitals and Aboriginal primary health services were highlighted as important. Additionally, stakeholders emphasised the importance of communication and collaboration across sectors, including DCP, particularly given mothers’ apprehension around their child being taken away. More focus on prevention and support, particularly for at-risk mothers, was seen as desirable, including intersectoral and interdisciplinary meetings and a holistic model of care that acknowledged upstream social determinants of health.

### Organisational

Participants across both hospital and primary health services suggested the need for more flexibility in service delivery and a greater focus on woman-centred care.

“I think we need to know what the women want. So, they should have some say in where they’re going to have their care, by whom and how often. We have standards of care where we say we want them to see us monthly for the first 36 weeks and then weekly until delivery at antenatal appointments and that, but we should be slightly more flexible with women who do come and go and move around. We should meet them where they’re at” (Reg 4H:6).

This approach was also reflected in participants’ responses from metropolitan, regional and remote sites who wanted direct feedback from mothers about the care they received in the hospital. This included inviting mothers to complete anonymous patient surveys before discharge as a strategy towards improving care (Reg 4H:7). Another participant felt more resourcing was needed around prevention.

“I just wish they would put more into the beginning of life stuff to help our parents build that stronger foundation and raise happy and healthy kids” (Metro 1C:1).

However, given the transient and sometimes unstable nature of living conditions for some women after discharge, some participants were worried that at-risk mothers might fall through the gaps. Discharge summaries not reaching primary health services promptly compromises postnatal care and increases the risk to mother and baby. Strategies to ensure mothers’ contact details are current and non-government primary health services receive birth notifications and discharge summaries promptly is essential.

While the trope of ‘I treat everyone the same’ was articulated by one midwife, a more flexible, midwife-led, culturally sensitive, woman-centred care seemed preferable. Some participants in a regional hospital advocated for midwifery-led care (Reg 4H:2) as well as more professional development for midwives (Reg 4H:8), including better courses on cultural safety (Reg 4H:7). Some felt that communicating with the mother in ways that were sensitive to her cultural background was more likely to build trust than adhering to a one-size-fits-all model of care. One participant reflected on the need for more culturally appropriate ‘yarning’ when giving information about breastfeeding. This would empower mothers rather than ‘box-ticking’; a ‘chat’ rather than ‘tell’ approach (Metro 2C:3).

Visiting Midwife Services (VMS) were also seen as an effective strategy to facilitate best practice in postnatal care, with one participant suggesting that all hospitals need a VMS (Metro 2C:1). These involve visits from a midwife up to 5 days post-discharge. Visits allow midwives to check in on mum and baby and provide an opportunity for information sharing. This can be particularly helpful when a mother opts for early discharge. One recently established VMS in a remote community was viewed positively by health providers and mothers for providing valuable support during the early postnatal period. Mothers discharged from a regional hospital could access the visiting midwife prior to being transferred to community health and the child health nurse. Some participants in a regional hospital advocated for midwifery-led care (Reg 4H:2) as well as more professional development for midwives (Reg 4H:8), including better courses on cultural safety (Reg 4H:7).

## Discussion

This research identified factors facilitating and impeding best practice in discharge planning, postnatal care and health education at a structural and organisational level. These are often beyond the control of individual health practitioners and may significantly impact the delivery of care, resulting in compromised maternal and infant health outcomes. The importance of continuity of care from hospital to home in the first 3 months of life was a key theme threading through the findings. Our findings suggest that this can be facilitated through adequate resourcing to support the time and staffing levels required to deliver optimal postnatal care and health education to mothers in the hospital. Other key aspects include training in culturally secure care, better follow up care post-discharge including improved coordination and dissemination of birth notifications and discharge summaries, and better communication and collaboration between the health and child protection sectors.

A recent scoping review on perinatal care for Aboriginal women indicated that health systems need to provide more resources to improve the delivery of culturally secure maternity care [23]. Various frameworks demonstrate how this can be achieved, and as noted by Coffin [24], the principles and practice of culturally secure care need to be demonstrated, not just by health practitioners but also by the health services. As stated in the CTG report, including Aboriginal women in decisions around design and delivery would be a positive step forward [34]. This was evident in the latest CTG campaign report highlighting the success of a partnership between the Mater Mothers’ Hospital in Brisbane and the Aboriginal and Torres Strait Islander Community Health Service established in 2016. This resulted in an Aboriginal community driven service offering culturally appropriate health and wellbeing care from conception till the child is 3 years old. Findings indicate a 50% reduction in premature births and an increase in antenatal attendance for participants on this program [35].

Our study adds to the body of research on postnatal care for mothers and Aboriginal babies [36]. It offers health services and policymakers an opportunity to review strengths and weaknesses in the current system around postnatal care for mothers of Aboriginal babies in Western Australia. Our findings reflect those in a South Australian study reporting the need to improve communication between hospitals and primary health care services to ensure ongoing engagement and follow up care of Aboriginal women in the postnatal period [37]. Child and Family Health Service (CaFHS) in South Australia worked closely with ACCHS and employed Aboriginal Cultural Consultants to help Aboriginal families participate in home visits. CaFHS nurses visit mothers and babies 1 to 4 weeks post-discharge from the hospital. However, those attending an Aboriginal health service for antenatal care were more likely to be visited by an Aboriginal Health Worker postnatally [37].

Evaluations of ACCHS programs to support Aboriginal mothers and Aboriginal babies in culturally safe ways have been documented, showing improved health outcomes [38, 39]. Key elements in successful programs include a holistic, strengths-based approach to meet the cultural, social and primary health care needs of women and their families. These programs included a two-way approach to care incorporating the western biomedical model with Aboriginal cultural ways of being, doing and knowing. This ensures Aboriginal women receive biomedical and cultural support throughout antenatal and postnatal care [40].

A home-visiting and advocacy programme delivered by an ACCHO in Victoria was seen positively by mothers and staff for promoting Aboriginal maternal and child health during the ante and postnatal stages of parenthood [41]. While key findings noted the importance of women’s connection to culture, it reflected challenges also identified in our study relating to staffing levels and the need for improved relationships with the Department of Child Protection.

Recurring themes between studies relate to the importance of culturally appropriate care where mothers feel welcome, safe and supported in caring for their Aboriginal babies. At a structural and organisational level, our findings reflect those of O’Donnell et al. [41] that suggest establishing and strengthening partnerships between services to improve the health and wellbeing outcomes for mothers and their Aboriginal babies. For example, better communication and collaboration between the maternal and child health and child protection sectors.

### Limitations

A limitation noted for this study was the inability to interview a broader mix of staff across the services involved. A broader mix of clinical and non-clinical staff such as allied health, administrative and senior management may have given us richer and more in-depth data. However, a strength of the study was the strong mix of staff providing close care of mothers at urban, regional and remote services.

## Conclusion

This study focusing on Western Australia adds to the current body of evidence with key structural and organisational factors that support, or compromise postnatal care delivered to mothers and their Aboriginal babies. Findings offer an opportunity to review current policies and practices for their effectiveness in underpinning good health outcomes in the first 3 months of life and ensuring adequate staff training in culturally safe care is applied to practice. Our findings demonstrate the need for mothers’ primary care providers to receive timely birth notifications and discharge summaries. Furthermore, the potential benefits of sectors working together to support mothers and babies at this vulnerable time will work towards improving maternal and child health outcomes.

## Data Availability

The datasets generated and/or analysed during the current study are not publicly available but are available from the corresponding author on reasonable request.

## Footnotes

1 ACCHO a primary health care service initiated and operated by the local Aboriginal community. It delivers holistic, comprehensive, and culturally appropriate health care to the local Aboriginal community and is governed through a locally elected Board of Management.
